# Long-Term Non-invasive Ventilation: Do Patients Aged Over 75 Years Differ From Younger Adults?

**DOI:** 10.3389/fmed.2020.556218

**Published:** 2020-11-11

**Authors:** Chloé Cantero, Dan Adler, Patrick Pasquina, Christophe Uldry, Bernard Egger, Maura Prella, Alain Bigin Younossian, Paola Soccal-Gasche, Jean-Louis Pépin, Jean-Paul Janssens

**Affiliations:** ^1^Division of Pulmonary Diseases, Geneva University Hospitals (HUG), Geneva, Switzerland; ^2^Faculty of Medicine, University of Geneva, Geneva, Switzerland; ^3^Division of Pulmonary Diseases and Pulmonary Rehabilitation Center, Rolle Hospital Rolle, Vaud, Switzerland; ^4^Division of Pulmonary Diseases, Lausanne University Hospital (CHUV), Lausanne, Switzerland; ^5^Division of Pulmonary Diseases and Intensive Care, La Tour Hospital, Geneva, Switzerland; ^6^HP2 Laboratory, Inserm U1042 Unit, University Grenoble Alps, Grenoble, France; ^7^EFCR Laboratory, Thorax and Vessels, Grenoble Alps University Hospital, Grenoble, France

**Keywords:** non-invasive ventilation, elderly, prevalence, compliance, chronic obstrucive pulmonary disease, obesity hypoventilation syndrome, ventilator settings

## Abstract

**Background:** Noninvasive ventilation (NIV) is accepted as standard of care for chronic hypercapnic respiratory failure (CHRF) and is being increasingly implemented in older subjects. However, little is known regarding the use of NIV on a long-term basis in the very old. The outcomes of this study were: 1/to report the proportion of patients ≥ 75 years old (elderly) among a large group of long-term NIV users and its trend since 2000; 2/to compare this population to a younger population (<75 years old) under long-term NIV in terms of diagnoses, comorbidities, anthropometric data, technical aspects, adherence to and efficiency of NIV.

**Methods:** In a cross-sectional analysis of a multicenter cohort study on patients with CHRF under NIV, diagnoses, comorbidities, technical aspects, adherence to and efficiency of NIV were compared between patients ≥ 75 and <75 years old (chi-square or Welch Student tests).

**Results:** Of a total of 489 patients under NIV, 151 patients (31%) were ≥ 75 years of age. Comorbidities such as systemic hypertension (86 vs. 60%, *p* < 0.001), chronic heart failure (30 vs. 18%, *p* = 0.005), and pulmonary hypertension (25 vs. 14%, *p* = 0.005) were more frequent in older subjects. In the older group, there was a trend for a higher prevalence of chronic obstructive pulmonary disease (COPD) (46 vs. 36%, *p* = 0.151) and a lower prevalence of neuromuscular diseases (NMD) (19 vs. 11%, *p* = 0.151), although not significant. Adherence to and efficacy of NIV were similar in both groups (daily use of ventilator: 437 vs. 419 min, *p* = 0.76; PaCO_2_: 5.8 vs. 5.9 kPa, *p* = 0.968). Unintentional leaks were slightly higher in the older group (1.8 vs. 0.6 L/min, *p* = 0.018).

**Conclusions:** In this cross-sectional study, one third of the population under NIV was ≥ 75 years old. Markers of efficacy of NIV, and adherence to treatment were similar when compared to younger subjects, confirming the feasibility of long-term NIV in the very old. Health-related quality of life was not assessed in this study and further research is needed to address this issue.

## Introduction

Long-term non-invasive ventilation (NIV) is an accepted treatment for chronic hypercapnic respiratory failure (CHRF). Since the beginning of long-term NIV in the mid 80's, its prevalence has increased substantially, with a European average of 6.6/10^5^ inhabitants in the Eurovent study (2000-1) (1) and reported values of 33–47/10^5^ inhabitants in recent data from Switzerland and Northern Europe (2018) (2). Long-term NIV is indicated in case of CHRF resulting from restrictive disorders (i.e., peripheral or central neurological disorders, myopathies, diseases affecting chest wall and/or pulmonary compliance such as kyphoscoliosis, or morbid obesity), obstructive disorders (such as chronic obstructive pulmonary disease) or sleep-related breathing disorders (3). Chronic obstructive pulmonary disease (COPD) is presently the most frequent cause of CHRF leading to NIV and tends to increase in older subjects (2). It is now well-accepted that NIV is efficient for treating acute episodes of hypercapnic respiratory failure (AEHRF) in older subjects by improving gas exchange and reducing respiratory work (3). However, little is known regarding the use of NIV on a long-term basis in the very old.

Advanced age *per se* may compromise the use of NIV in this population because of functional decline, cognitive impairment, frailty, and other causes of disability including neurological or rheumatological impairment. Appropriate positioning of interface, and thus unintentional leaks and treatment efficacy, could also be a problem, as well as skin sores. Furthermore, a French multicentric cohort study suggested that, although NIV improves arterial blood gas (ABG) and sleepiness in subjects ≥75 years of age, it does not improve health-related quality of life (HRQL, measured by the SF-36), as opposed to what is seen in younger subjects (4). Indeed, there are to date very few published reports of long-term NIV in the very old (4–7).

We recently conducted a comprehensive survey of NIV in the Cantons of Geneva and Vaud (≈ 1,300,000 inhabitants) (2). The outcomes of this study were: 1/to document the proportion of patients ≥75 years old (elderly) among a large group of long-term NIV users and its trend since 2000; and 2/ to compare this population to younger subjects (<75 years old) under long-term NIV in terms of diagnoses, comorbidities, technical aspects (i.e., choice of devices, modes, settings, interfaces, unintentional leaks), adherence to and efficiency of NIV. HRQL was not assessed in this study.

## Patients and Methods

A detailed description of the methodology of this study has been recently published (2). Briefly, this analysis was based on a cross-sectional observational study performed in 2018 and including all patients under NIV in our area (Cantons of Geneva and Vaud, that is, a population of 1,288,378 inhabitants). Identification, screening, and data collection were performed by two investigators between June 1, 2016 and July 10, 2018.

Ethical approval was granted by the Cantonal Commission for Research Ethics (CCER) in Geneva, Switzerland (n°PB_2016-00925/15-275) in agreement with the amended Declaration of Helsinki. Trial was registered at clinicaltrials.gov (N°: NCT04054570).

The present study focuses exclusively on patients treated by pressure-cycled, multimodal and volume-cycled ventilators at home or in a long-term care facility (not a hospital) for ≥3 months. Patients were excluded if they refused data collection regarding their long-term NIV, or if their pulmonologist refused to participate in the study.

### Outcomes

The outcomes of this study were: 1/to report the proportion of patients ≥75 years old (elderly) in a comprehensive database of long-term NIV users in the Cantons of Geneva and Vaud; 2/to compare these results to a similar study performed earlier in our area (data from 2000); 3/to provide a detailed description of diagnoses, comorbidities, technical aspects (i.e.: choice of devices, modes, settings, interfaces, unintentional leaks), adherence to and efficiency of NIV and compare these results with similar data collected from younger subjects (<75 years old) under long-term NIV in the same cross-sectional cohort study.

### Data Collected

Anthropometric data, diagnoses leading to NIV, major comorbidities, pulmonary function tests, ABG, nocturnal pulse oximetry, technical aspects of NIV (i.e.: choice of devices, modes, settings, interfaces, unintentional leaks), adherence and relevant items from reports downloaded from device software were collected from medical records. Availability of recent pulmonary function tests, ABG and nocturnal pulse oximetry depended on “real-life” follow-up procedures and medical records. Data recorded were the most recent measurements performed within the 12 months prior to data collection. Tests which had not been performed within the previous 12 months were considered as missing data. We also recorded whether NIV was initiated in an acute setting, or electively, and as an outpatient vs. an inpatient setting (hospital ward). Prevalence was compared to values published in 2000 from the same area.

### Diagnostic Categories

For all patients, indication for implementing NIV was based on the 1999 Consensus conference report. There is no “*a priori*” attitude regarding NIV in older subjects in our area and therefore the selection of patients is not age dependent.

### Statistical Analyses

Patients' characteristics, efficiency and technical aspects of NIV were described overall and by age group. Qualitative data were described as simple frequency and percentage, quantitative data were described as median (first quartile, third quartile). Qualitative data were compared between age groups using chi-square tests. Quantitative data were compared between age groups using Welch *t* tests. Statistical significance was assessed at a two-sided 0.05 alpha level for all analyses. No correction for multiple testing was applied. Analyses were performed on R software (R Foundation for Statistical Computing, Vienna, Austria).

## Results

### Prevalence

The proportion of patients under long-term NIV aged ≥ 75 years of age increased in our area from 17% in 2000 (8) to 31% in 2018 (151/489) (2). In 2000 (8), 8.4% of patients (13/154) under long-term NIV had their treatment initiated at ≥ 75 years and 27/154 (17.5%) of NIV patients were aged ≥ 75 years. In 2018, 109/489 (22%) were ≥75 years of age when NIV was started, and prevalence of subjects aged ≥ 75 was 31% (151/489) (2).

### Population Characteristics

**Figure 1:**
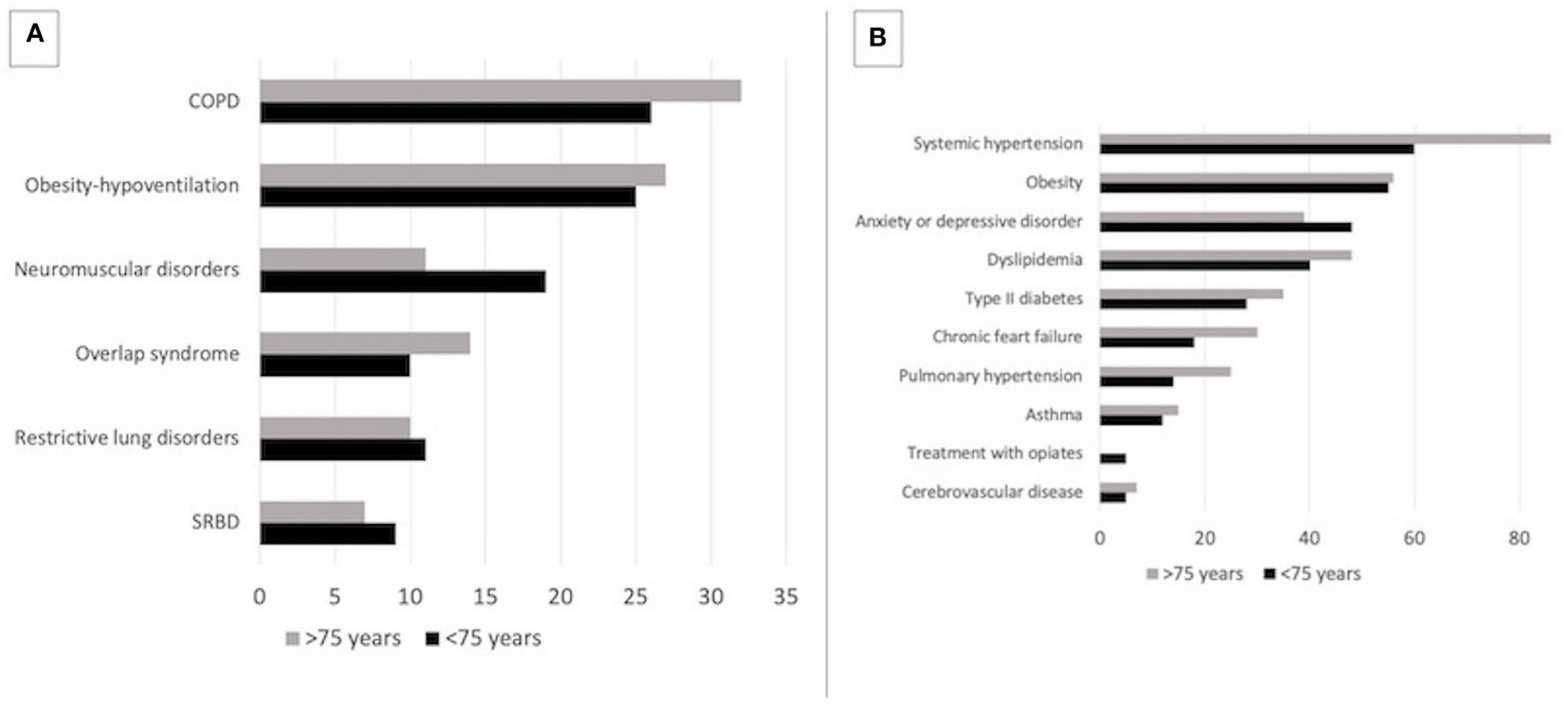
**(A)** Distribution of indications for long-term NIV according to age group. **(B)** Frequencies of comorbidities according to age group. COPD, Chronic obstructive pulmonary disease; SRBD, Sleep-related breathing disorders; Overlap syndrome, association of COPD and obstructive sleep apnea syndrome.

Table 1 provides the basic characteristics of the study population by age group. Patients had been under NIV for a similar length of time (median value of ≈ 3 years). In the older group, there was a trend for a higher prevalence of chronic obstructive pulmonary disease (COPD) (46 vs. 36%, *p* = 0.151) and a lower prevalence of neuromuscular diseases (NMD) (19 vs. 11%, *p* = 0.151), although non-significant (Figure 1A). Body mass index (BMI) was similar in both groups (Table 1, *p* = 0.26). Older subjects had significantly more comorbidities (*p* = 0.001, Table 1) and comorbidities such as systemic hypertension (86 vs. 60%, *p* < 0.001), chronic heart failure (30 vs. 18%, *p* = 0.005), or pulmonary hypertension (25 vs. 14%, *p* = 0.005) were more frequently reported in the elderly (Figure 1B). Conversely, treatment of central sleep apnea due to opioids was less frequent in older subjects (0 vs. 5%, *p* = 0.011, Figure 1B). Modalities of initiation of NIV were similar in both groups: electively (48 vs. 55%, *p* = 0.151) vs after an AEHRF (52 vs. 45%, *p* = 0.151), outpatient (12 vs. 17%, *p* = 0.191) vs. inpatient setting (88 vs. 83%, *p* = 0.191) (Table 1).

**Table 1:** Characteristics of study population according to age group.

	**All patients 489 (100)**	**<75 years 338 (69)**	**≥ 75 years 151 (31)**	***P*-value**
**Population characteristics**				
Gender (male)	272 (56)	203 (60)	69 (46)	0.004
Age (years)	71 (59; 77)	64 (52; 71)	80 (78; 83)	NA
Age (years)*	5 to 94	5 to 74	75 to 94	NA
Age when NIV started (years)	65 (53; 73)	59 (48; 66)	76 (74; 80)	<0.001
Body-mass index (kg/m^2^) *Missing data* (*n*)	31 (24; 39) 1	31 (23; 40) 1	31 (24; 37) 0	0.26
Number of comorbidities *Missing data* (*n*)	3 (2; 4) *1*	3 (1; 4) *1*	3 (2; 5) *0*	0.001
Time spent under NIV (months)	39 (14; 73)	40 (14; 74)	36 (15; 69)	0.235
**Initiation of NIV**				
Electively *Missing data* (*n*)	247 (53) *22*	179 (55) *14*	68 (48) *8*	0.151
After an AEHRF *Missing data* (*n*)	220 (47) *22*	145 (45) *14*	75 (52) *8*	
Inpatient setting *Missing data* (*n*)	400 (84) *16*	273 (83) *9*	127 (88) *7*	0.191
Outpatient setting **Missing data** (*n*)	73 (16) *16*	56 (17) *9*	17 (12) *7*	
**Diagnostic groups**				
COPD	192 (39)	123 (36)	69 (46)	0.151
Obesity-hypoventilation syndrome	127 (26)	86 (25)	41 (27)	
Neuromuscular disorders	79 (16)	63 (19)	16 (11)	
Restrictive lung disorders	49 (10)	34 (10)	15 (10)	
Sleep-related breathing disorders	42 (9)	32 (9)	10 (7)	

*P-values refer to Chi-square tests or Welch Student tests*.

*number (=n) of missing data, corresponds to the number of patients without the values of interest*.

Values listed are the most recent values obtained. Values expressed as median (interquartile range) or n (%) unless specified otherwise. *Range.

*AEHRF, acute episode of hypercapnic respiratory failure; COPD, chronic obstructive pulmonary disease; NA, not applicable; NIV, non-invasive ventilation*.

### Interfaces, Adjuncts to and Efficiency of NIV

Table 2 shows the impact of NIV on ABG (values are without NIV) and nocturnal pulse oximetry (NPO, overnight under NIV). Missing values reflect “real life” availability of tests during the study process. Taking into account this caveat, correction of ABG and NPO was similar in both groups (Table 2). Choice of interfaces was similar (Table 3, *p* = 0.170), with a very high proportion of facial masks in both age groups (79 vs. 72%, *p* = 0.170). Use of supplemental oxygen was more frequent in the older group (54 vs. 36%, *p* < 0.001, Table 3). Use of humidifiers was similar in both groups (75 vs. 70%, *p* = 0.386).

**Table 2:** Arterial blood gases without NIV and nocturnal pulse oximetry under NIV according to age group.

	**All patients 372 (100)**	**<75 years 251 (67.5)**	**≥ 75 years 121 (32.5)**	***P*-value**
**Arterial blood gases without NIV**				
pH	7.40 (7.38; 7.43)	7.40 (7.38; 7.42)	7.40 (7.38; 7.43)	0.546
PaCO_2_ (kPa)	5.8 (5.3; 6.5)	5.9 (5.2; 6.5)	5.8 (5.3; 6.5)	0.968
HCO_3_ (mmol/L)	26.9 (24.8; 30)	26.8 (24.6; 29.6)	27.9 (25.3; 30.5)	0.183
PaO_2_ (kPa)^*^	9 (8; 9.9)	9.1 (8; 10.1)	9.1 (8.1; 10.0)	0.856
SaO_2_ (%)^*^	94 (92; 96)	94 (92; 96)	94 (91; 96)	0.536
	**All patients 197 (100)**	**<75 years 138 (70)**	**≥75 years 59 (30)**	***p*****-value**
**Nocturnal pulse oximetry under NIV**				
Mean SpO_2_ (%)	92 (90; 94)	93 (90; 94)	91 (91; 94)	0.184
Time with SpO_2_ <90% (%)	6 (0.4; 34.3)	6 (0.1; 34.2)	5.3 (0.7; 34.5)	0.636
ODI ≥ 3% (events/hour)	7.3 (3.2; 13.8)	7.5 (3.2; 14)	7.3 (3.4; 13.5)	0.538
	**All patients 353 (100)**	**<75 years 242 (68.5)**	**≥** **75 years 111 (31.5)**	***p*****-value**
**Pulmonary function tests**				
FEV_1_ (% predicted)	46 (31; 64)	44 (29; 62)	46 (35; 64)	0.192
FVC (% predicted)	62 (45; 75)	60 (44; 74)	65 (48; 76)	0.129
FEV_1_/FVC (%)	83 (63; 98)	83 (64; 98)	84 (63; 97)	0.742

*P-values refer to Chi-square tests or Welch Student tests*.

*Values listed are the most recent values obtained*.

*Values expressed as median (interquartile range) or n (%) unless specified otherwise*.

* *PaO_2_ and SaO_2_ are room air values (PaO_2_: n = 92; SaO_2_: n = 89)*.

*FEV_1_, forced expiratory volume in 1 s; FVC, forced vital capacity; ${\text{HCO}}_{3}^{-}$: bicarbonates; NIV: non-invasive ventilation; ODI, oxygen desaturation index (≥3%); PaCO_2_, arterial partial pressure of carbon dioxide; PaO_2_, arterial partial pressure of oxygen; SaO_2_, arterial oxygen saturation; SpO_2_, pulse oximeter oxygen saturation*.

**Table 3:** Devices used for long-term NIV with modes, interfaces, and adjuncts to NIV according to age group.

	**All patients 489 (100)**	**<75 years 338 (69)**	**≥75 years 151 (31)**	***P*-value**
**Bi-level positive pressure ventilators (*****missing data*****:** ***n*** **=** **3)**				
ST mode	407 (83)	272 (80)	135 (89)	0.150
Auto-titrating modes	68 (14)	52 (15)	16 (11)	
**Multimodal ventilator modes (*****missing data*****:** ***n*** **=** **1)**				
VAC, PC or PS	10 (2)	10 (2)	0 (0)	NA
**Interfaces**				
Facial	359 (73)	240 (72)	119 (79)	0.170
Nasal	91 (19)	66 (20)	25 (17)	
Nasal pillows	40 (8)	31 (9)	7 (5)	
*Missing data* (*n*)	*3*	*3*	*0*	
**Other adjuncts to NIV**				
Humidifiers *Missing data* (*n*)	350 (72) *2*	237 (70) *2*	113 (75) *0*	0.386
Oxygen	196 (40)	123 (36)	81 (54)	<0.001

*P-values refer to Chi-square tests or Welch Student tests*.

*number (=n) of missing data, corresponds to the number of patients without the values of interest*.

*Values listed are the most recent values obtained*.

*Values expressed as median (interquartile range) or n (%) unless specified otherwise*.

*NA, not applicable; NIV, non-invasive ventilation; PC, pressure control; PS, pressure support; ST, spontaneous-timed; VAC, volume assist-control*.

### Devices, Settings, Unintentional Leaks and Adherence

Use of bi-level positive pressure ventilators (BPPV) in a spontaneous/timed (S/T) mode was by far the most frequent modality of NIV (89 vs. 80%, *p* = 0.150, Table 3). None of the older subjects used multimodal devices or volume-cycled modes (Table 3). Table 4 shows basic ventilator settings for BPPV devices in an S/T mode (i.e., 83% of the whole population, 89% of those aged ≥ 75 years). In the elderly, 11% used auto-titrating BPPV devices. No significant difference was noted in pressure settings, back-up respiratory rate or residual respiratory events estimated by ventilator software (Table 4). Adherence to NIV (average time spent under NIV) was similar in both groups (Table 4). The percentage of patients using their device <03:30 h was 7% in the older group and 9% in the younger subjects (median values). However, unintentional leaks (median and 95^th^ centile values) were significantly higher in older subjects (Table 4).

**Table 4:** Settings and data provided from ventilator software for bi-level positive pressure ventilators in ST mode according to age group.

	**All patients 407 (100)**	**<75 years 272 (67)**	**≥75 years 135 (33)**	***P*-value**
**Settings for bi-level positive pressure ventilators in ST mode**				
IPAP (cmH_2_O)	18 (16; 21)	18 (16; 22)	18 (16; 21)	0.93
EPAP (cmH_2_O)	7 (5; 10)	7 (5; 10)	7 (6; 9)	0.27
BURR (cycles/min)	14 (12; 17)	14 (12; 17)	15 (12; 17)	0.08
**Data from ventilator software**				
Median leaks^*^ (L/min)	1.2 (0; 7.2)	0.6 (0; 7)	1.8 (0; 8.4)	0.018
Median leaks^*^ 95th centile (L/min)	14.4 (3; 30)	12.6 (2.3; 28.3)	16.8 (6; 33)	0.028
AHI (events/hour)	1.5 (0.4; 4.1)	1.3 (0.3; 3.6)	2.1 (0.6; 5)	0.138
**Adherence**				
Daily use of ventilator (min)	428 (310; 532)	419 (304; 534)	437 (321; 529)	0.76

*P-values refer to Chi-square tests or Welch Student tests*.

*Values listed are the most recent values obtained*.

*Values expressed as median (interquartile range) or n (%) unless specified otherwise*.

* *Unintentional leaks*.

*AHI, apnea-hypopnea index; BURR, back-up respiratory rate; EPAP, expiratory positive airway pressure; IPAP, inspiratory positive airway pressure; ST, spontaneous-timed*.

## Discussion

This cross-sectional observational study shows that 1/close to one third of the population under NIV is presently aged over 75 years of age in this study area: this is a substantial increase since our previous survey, 18 years ago (8); 2/disorders leading to CHRF and NIV did not differ significantly, with a trend for a higher proportion of COPD in the older group, and a lower representation of NMD; 3/efficiency of NIV (daytime ABG, nocturnal pulse oximetry, residual respiratory events) was similar in both groups, but unintentional leaks were slightly although significantly more important in older subjects; and importantly 4/adherence to treatment did not differ between groups.

The first study focusing on NIV in older subjects in our area had identified 6 patients between 1994 and 1996 in an on-going cohort study in whom NIV had been initiated at or after 75 years of age (8, 9). Efficiency of NIV, tolerance and adherence to NIV and HRQL were all very satisfactory. What was at that time a very rare occurrence is presently standard practice with close to one third of patients under NIV being aged ≥ 75 years. Interestingly, this prevalence is exactly the same as that reported in a French multicentric cohort study (2009-14; *n* = 264) by Tissot et al. (4). Similar trends are also reported in countries which have a national register of NIV such as Norway (Norwegian national registry for long-term NIV) and Sweden (Swedevox data). In 2007 already, 12% of the population on long-term NIV in Sweden was aged over 75 (10).

In the present study, there was a trend for COPD to be more frequent and neuromuscular diseases to be less frequent in older subjects (Figure 1A): prevalence of COPD increases with age and is thus a more frequent cause of AEHRF and CHRF (11, 12). Conversely, NMD is rarely an indication for long-term NIV (albeit for ALS) in the elderly and subjects with NMD leading to NIV rarely reach the age of 75. Patients with “historical” causes of CHRF such as sequelae of tuberculosis or post-polio syndrome, have now almost disappeared. Other causes of CHRF in the older population such as chronic heart failure, cerebrovascular disease, or interstitial lung disease seldom lead to long-term NIV (3). Interestingly, the obesity epidemic— and thus obesity hypoventilation syndrome (OHS)—also affects the very old: 27% of our older patients are under NIV for OHS! OHS represented 52% of the elderly population in the French cohort study by Tissot et al. (4). Earlier studies reported lower figures for OHS in this age group (14–20%) (4–7), suggesting that the increase in OHS as a cause of CHRF noted in younger adults may also involve the older population.

Adherence to treatment was excellent in older subjects without any significant difference compared to younger subjects, confirming previous reports that age *per se* does not seem to adversely affect adherence (4, 6, 7). The percentage of subjects using their NIV insufficiently (arbitrarily defined as <03:30 h/day) was 7% in the older group (vs. 9% in younger subjects). However, the cross-sectional structure of our study does not allow us to comment on the discontinuation rate of NIV. The fact that these patients were on long-term NIV for a median duration of 3 years suggests that the treatment was well-tolerated, considered acceptable, and did not adversely affect HRQL.

Because of the cross-sectional nature of this study, we have no information on survival: however, as stated, patients aged ≥ 75 had been under NIV for a median of 3 years, which shows that prolonged acceptance with good adherence is possible at this age. A few studies provide encouraging figures in subjects aged ≥ 75 under long-term NIV: Laub et al. (13) and Duiverman et al. (14) noted a 5-year survival close to 50% in this age group, while Farrero et al. (5) reported a median survival of 58.5 month in non-ALS older patients.

Technical aspects of NIV did not show any major difference in the older group. All patients aged over 75 used BPPV in an S/T mode or auto-titrating devices, following the trend described for all patients since the late 90's (8). None had a volume-cycled device. Pressure settings and choice of interface (predominantly facial masks in our area) were similar in both age groups. Noteworthy are the very satisfactory results on control of ABG and nocturnal pulse oximetry: all patients were initially hypercapnic whether in a stable condition or after an AEHRF. Unintentional leaks increased with aging: this was expected as a consequence of mispositioning of interface and/or changes in texture of facial subcutaneous tissue associated with aging. However, the median and peak values obtained in older subjects are well within what is clinically acceptable, and in most cases did not compromise the efficiency of NIV.

### Study Limitations

There are several limitations to this study. 1/This cross-sectional study describes a selected population which have accepted and adapted to NIV: we do not have information as to prior dropouts or refusals. However, the large group of older subjects described supports the idea that long-term NIV is feasible on a long-term basis in the very old, with a similar efficacy in terms of correction of ABG and nocturnal SpO_2_ as in younger subjects; 2/Missing data reflect the “real life” nature of this study; 3/These data are related to the socioeconomic conditions, demographics, and epidemiology prevailing in Switzerland and may not reflect findings in different geographic, economic or ethnic settings; they show however that advanced age *per se* is not a contra-indication to long-term NIV; 4/We could not provide information on burden for care-givers in older subjects: this must be further assessed, since this may be a critical factor in a population which is already affected by several comorbidities.

### Clinical and Research Implications

Based on the various results of our study, age itself alone should not be a criterion of exclusion if long-term NIV is considered. However, impact on HRQL and burden placed on caregivers requires further studies.

## Conclusions

In this observational study of patients on long-term NIV, patients aged over 75 years of age represented almost one third of the population treated and had the same benefit in terms of correction of ABG, nocturnal pulse oximetry, as younger adults in the same area. Adherence, residual respiratory events were similar to that of younger subjects, and quite satisfactory. Although unintentional leaks were increased in older subjects, this was within acceptable median and peak values. COPD was the most important diagnostic group, and OHS seems to be increasing. These data confirm the feasibility of long-term NIV in the very old.

## Data Availability Statement

The raw data supporting the conclusions of this article will be made available by the authors, without undue reservation.

## Ethics Statement

The studies involving human participants were reviewed and approved by the Cantonal Commission for Research Ethics (CCER) in Geneva, Switzerland (no. PB_2016-00925/15-275) in agreement with the amended Declaration of Helsinki. The patients/participants provided their written informed consent to participate in this study.

## Author Contributions

CC, PP, and J-PJ contributed to the conception and design of the study. CC and PP organized the database. CC and J-PJ wrote the first draft of the manuscript. CC, PP, DA, and J-PJ wrote sections of the manuscript. All authors contributed to manuscript revision and read and approved the submitted version.

## Conflict of Interest

The authors declare that the research was conducted in the absence of any commercial or financial relationships that could be construed as a potential conflict of interest.
